# Vision-related quality of life and psychological status in Chinese women with Sjogren’s syndrome dry eye: a case-control study

**DOI:** 10.1186/s12905-016-0353-z

**Published:** 2016-12-12

**Authors:** Yuqiu Zhang, Tong Lin, Alice Jiang, Naiqing Zhao, Lan Gong

**Affiliations:** 1 Department of Ophthalmology, Eye & ENT Hospital of Fudan University, No. 83 Fenyang Road, Shanghai, 200031 People’s Republic of China; 2 School of Medicine & Public Health, University of Wisconsin, Madison, USA; 3 Department of Biostatistics, School of Public Health, Fudan University, Shanghai, People’s Republic of China; 4 Key Laboratory of myopia, Ministry of Health, Shanghai, People’s Republic of China

**Keywords:** Sjogren’s syndrome, Dry eye, Vision-related quality of life, Anxiety, Depression

## Abstract

**Background:**

Sjogren’s syndrome dry eye (SSDE) mainly affects middle-aged women and can negatively affect women’s psychological and social functioning. However, little is known about the correlation between vision-related quality of life (VR-QoL) and psychological status for women with SSDE. We therefore examined VR-QoL and psychological status in two groups of Chinese women: an SSDE group and a non-SSDE group. We also explored the associations between VR-QoL scores, sociodemographic measures, ophthalmologic parameters, and psychological status in women with SSDE.

**Methods:**

The case-control study recruited 30 female outpatients with SSDE and 30 without SSDE from the Eye and Ear, Nose, and Throat (ENT) Hospital of Fudan University. Demographic and ophthalmologic data were collected from all participants. Ophthalmologic examinations included best-corrected visual acuity (BCVA), corneal fluorescein staining (CFS), tear break-up time (TBUT) and Schirmer test. Data collected using the National Eye Institute’s Visual Function Questionnaire (NEI-VFQ) and Ocular Surface Disease Index (OSDI) survey instruments were analyzed to identify potential differences in VR-QoL between the SSDE group and the non-SSDE group. We also used the Zung Self-Rating Anxiety and Self-Rating Depression Scales (SAS and SDS) to determine psychological status in both groups.

**Results:**

The SSDE group scored significantly lower than the non-SSDE group on the NEI-VFQ subscales of general health, general vision, and long-distance vision activities (all *p* < 0.05). The SSDE group achieved a significantly higher ocular symptoms score compared with the control group (*p* = 0.0256). The SAS and SDS scores of the SSDE group were significantly higher than the non-SSDE group (*p* = 0.0072 and 0.0162, respectively). The prevalence of anxiety and depression in the SSDE group was significantly higher than the non-SSDE group (*p* = 0.0240 and 0.0200, respectively). Nine of twelve NEI-VFQ subscales were negatively correlated with SAS/SDS scores (all *p* values were <0.05). The exceptions were social function, color vision and peripheral vision. The composite OSDI score and its three subscale scores for the women in the SSDE group were all positively correlated with overall SAS/SDS scores (all *p* values were <0.05).

**Conclusions:**

Both VR-QoL and psychological status were significantly worse in SSDE group than in the non-SSDE group. The VR-QoL of women with SSDE had a negative correlation with their anxiety and depression levels.

## Background

The second most common autoimmune disease, Sjogren’s syndrome (SS) mainly involves the exocrine glands, especially the salivary and tear-producing glands [1], with an estimated prevalence of 0.1–4.8% [2–4]. The syndrome exhibits a female-to-male ratio of 9:1 and mainly affects middle-aged women [3]. In addition to physical loss of function of the exocrine glands, SS can negatively affect women’s psychological and social functioning [5, 6]. In a previous study, Inal et al. reported that SS tended to impair both health-related quality of life and psychological wellbeing [7]. Women with SS often complain about dry eye symptoms as a result of severe keratoconjunctivitis. Common ocular symptoms of Sjögren syndrome dry eye (SSDE) include burning, itchiness, photaesthesia, pain in the eyes, blurred vision, or tired eyes [8, 9]. In patients with SSDE, chronic ocular inflammatory lesions involve injury to the epithelial cells of the conjunctiva and cornea. [10] As a result, ocular manifestations in SSDE are commonly more severe than in non-SSDE conditions [11, 12]. Patients with SSDE, who often continue to experience ocular symptoms despite extended use of palliative treatments, such as artificial tears, punctual occlusion, and acupuncture [13], may be prone to excessive medical behavior and high levels of mental stress. In a previous study, we found that impaired vision-related quality of life (VR-QoL) in dry eye patients without SSDE could be correlated with psychological disturbances [14]. We therefore hypothesized that SS would be characterized by severe dry eye conditions as well as systemic disease factors, which could have a significant additional impact on women’s VR-QoL and psychological status, when compared with non-SSDE conditions. To the best of our knowledge, few studies have yet been designed to investigate interactions between the clinical characteristics of women with SSDE, their VR-QoL, and their psychological status.

As a result, our study focused on VR-QoL and psychological status in Chinese women both with and without SSDE, using questionnaires including National Eye Institute Visual Function Questionnaire (NEI-VFQ), Ocular Surface Disease Index (OSDI), Zung Self Rating Anxiety Scales (SAS) and Zung Self Rating Depression Scales (SDS). We also examined the associations between VR-QoL scores, sociodemographic measures, clinical parameters, and psychological status for women with SSDE. Our findings may be important in identifying the effect of SSDE on women’s VR-QoL and in evaluating the psychological disturbances that they experience. It may also suggest new ways to enhance quality of life among these patients.

## Methods

### Study design and participants

We adopted an outpatient-based case-control study design. This study recruited 30 women outpatients with SSDE and 30 without SSDE, who attended the Department of Ophthalmology at the Eye and ENT Hospital of Fudan University from September 2012 to December 2012. All SSDE participants fulfilled the American-European Consensus Group classification criteria [15]. Of the patients with SSDE, 22 met the diagnostic criteria for primary SS, and eight for secondary SS (of these, six had concurrent rheumatoid arthritis and two had concurrent systemic lupus erythematosus). Participants in the non-SSDE group had a similar age as participants in the SSDE group. All the participants met the following criteria: a Schirmer test value of less than 10 mm/5 min, a tear break-up time (TBUT) of less than 10 s in each eye, and one or more moderate dry eye related symptom (dryness, foreign body sensation, burning, asthenopia, blurred vision, or a sensation of stabbing pain). The study eye was defined as the eye with the lowest baseline Schirmer test. If both eyes had the same Schirmer test value, the right eye was selected as the study eye. Subjective symptoms were scored on a 4-point scale, with scores of 0 to 3 (with three representing the most severe symptoms). Symptoms with a score of more than two were defined as being at a moderate level. All participants had best corrected visual acuity (BCVA) of 20/40 or better in each eye. We reviewed the medical history of all the participants to exclude patients who had severe psychiatric disorders such as mania, schizophrenia, or long-term severe depression and anxiety caused by other diseases. Participants who had had topical ocular treatment (ocular laser or surgical), who had worn contact lenses within the past 6 months, or who suffered from other ocular diseases, such as glaucoma or cataract, were also excluded.

### Ethics statement

This study was performed according to the principles of the Declaration of Helsinki and was approved by the Ethics Committee of the Eye and ENT Hospital of Fudan University. All recruited participants read and signed an information and consent form before participating in the study.

### Study procedure and setting

Demographic and clinical data were collected from all participants. Demographic data included name, age, educational level, marital status, household income level, and disease duration. Clinical features included BCVA, corneal fluorescein staining (CFS), TBUT and Schirmer test without anesthesia (S1T). All patients completed the NEI-VFQ, OSDI, SAS and SDS questionnaires by themselves after the ophthalmic examinations. In order to minimize the effect of questionnaire order, all patients completed the questionnaires in the same order with the NEI-VFQ first, followed by the OSDI, SAS, and SDS questionnaires. To alleviate the task burden of these questionnaires, we ensured that all patients had sufficient time and introductory information to complete the questionnaires. In order to minimize the therapeutic effect, no treatment was given to the participants on the visit day. All participants were tested between 9:00 and 12:00 am in a consulting room in which central air conditioning and humidifiers controlled both temperature (15–25 °C) and humidity (30–50%). To avoid reflex tearing, no light other than the room light was used in the consulting room.

### Data collection instruments

#### Ocular examinations

All ophthalmologic examinations were performed by the same ophthalmologist. The BCVA was evaluated with a LogMAR visual acuity chart. The S1T was used to measure tear production, by inserting a dry Schirmer test strip (Jingming, Tianjing, China) over the outer third of the lower eyelid margin. The distance that the tears had traveled along the test strip at 5 min was recorded as the S1T score. Potential scores range from 0 to 30 mm, with lower scores indicating reduced function. After a 30-min rest, TBUT was measured by instilling fluorescein into the lower fornix with a damp fluorescein strip (Jingming, Tianjing, China). The patient was then asked to blink several times. Using cobalt blue filter and slit lamp biomicroscopy, the time required to observe the first area of tear film break up after a complete blink was recorded as the TBUT. The test was repeated three times and the average of the three measurements was calculated. CFS was graded on a scale of 0–3 points (none to severe) in each quadrant (total points, 0–12).

#### VR-QoL assessment

Data collected from the NEI-VFQ and OSDI survey instruments were analyzed to identify potential differences in VR-QoL between the SSDE and non-SSDE groups. The NEI-VFQ questionnaire was developed to evaluate VR-QoL in patients with ocular diseases, and to test the psychometric properties of diseases that cause visual disturbances [16]. The 25-item NEI-VFQ has been proven to evaluate the quality of life in patients with non-SSDE in previous studies [14, 17]. The Chinese version has been validated according to established criteria for reliability and validity [18]. This version contains 39 items and 12 domains or subscales. The 12 domains include general health, general vision, ocular pain, short-distance vision activities, long-distance vision activities, social functioning, mental health, role difficulties, dependency, driving difficulties, color vision, and peripheral vision. The questionnaire includes statements about problems, which involve patients’ vision or feelings at the current time. A standard algorithm was used to calculate the scale scores. Scores ranged from 0 to 100, with higher scores indicating better function. Eleven of the twelve scale scores (excluding the general health item) were averaged to yield a composite score [19].

The OSDI, developed by the Outcomes Research Group at Allergan Inc. (Irvine, Calif), is a 12-item questionnaire that assesses symptoms of ocular irritation consistent with dry eye disease and their impact on vision-related functioning [20]. On entry to the study, each participant completed an OSDI questionnaire that contained 12 questions to evaluate the character and severity of dry eye symptoms, including vision-related function, ocular symptoms, and environmental triggers encountered over the past week. The participants were asked to score the frequency of symptoms from none to all the time. The composite OSDI score ranges from 0 to 100, with higher scores indicating more severe symptoms. The OSDI has been validated in Chinese populations and has been widely used and accepted by ophthalmologists in China.

#### Psychometric measures

The SAS questionnaire is a 20-item, four-point Likert scale self-report assessment for the presence and severity of affective symptoms and somatic components of anxiety during the previous week [21]. Fifteen of the items express negative experiences or symptoms (e.g., “I am afraid for no reason at all”) and five express positive experiences and are reversed (e.g., “I feel that everything is all right and nothing bad will happen”). The SDS questionnaire is a 20-item four-point Likert scale self-report assessment for depression during the previous week [22]. Ten of the items express negative experiences or symptoms (e.g., “I have crying spells or feel like it”) and ten express positive experiences and are reversed (e.g., “My life is pretty full”). Each question was scored on a scale of 4 (1, none or a little of the time; 2, some of the time; 3, much of the time; and 4, most of the time). The raw total scores were converted to a 100-point scale by multiplying by 1.25. Participants who scored more than 50 on the SAS and SDS scales were considered to have symptoms of anxiety and depression, respectively [23]. The SAS and SDS scores, as well as the prevalence of anxiety and depression, were used to determine psychological status for both groups.

### Data management and analysis

All data have been analyzed with the Statistical Package for the Social Sciences (SPSS, version 18.0, SPSS Inc., Chicago, Illinois, USA). Data are expressed as mean ± standard deviation. Mann-Whitney U tests were used to compare continuous variables. The categorical variables were analyzed using the chi-square test. Spearman’s rank correlation test was applied to analyze the relationship between the NEI-VFQ/OSDI and SAS/SDS scores, and socio-demographic and clinical parameters. Regression analyses were performed to examine how the various demographic and clinical variables and the VR-QoL scores contributed to the depression and anxiety scores in women with SSDE. *P* values >0.05 were accepted as significant.

## Results

### Patient characteristics

Demographic and ophthalmologic data for the SSDE and non-SSDE groups are presented in Table 1. The two groups were comparable with regards to matched age, marital status, household income levels, education levels and duration of dry eye symptoms (all *p* values >0.05). As expected, a significant difference was detected between the groups for ophthalmologic data, including TBUT, S1T and CFS (all *p* values were <0.05), with more severe ocular symptoms in the SSDE group than in the non-SSDE group.

**Table 1:** Demographic characteristics and ophthalmologic data in the SSDE and non-SSDE groups

	SSDE (*n* = 30)	Non-SSDE (*n* = 30)	*P* value
Age (years)	46.8 ± 11.1	49.8 ± 10.2	0.2864
Marital status, n (%)			0.6120
Married	29 (96.7)	27 (90.0)	
Single	1 (3.3)	3 (10.0)	
Household income, n (%)			0.6940
Low (< RMB3000)	19 (63.3)	22 (73.3)	
Moderate (RMB3000–5000)	5 (16.7)	4 (13.3)	
High (>RMB5000)	6 (20.0)	4 (13.3)	
Education level, n (%)			0.5530
Primary school	1 (3.3)	0 (0)	
High school	12 (40)	14 (46.7)	
University	17 (56.7)	16 (53.3)	
In use of tear supplements, n (%)	23 (76.7)	19 (63.3)	0.3990
Duration of disease (years)	1.75 ± 1.80	1.50 ± 1.60	0.6225
BCVA(logMAR)	0.10 ± 0.12	0.10 ± 0.10	0.8848
TBUT (second)	3.10 ± 2.64	4.67 ± 2.27	0.0098*
S1T (mm/5 min)	1.30 ± 1.55	2.57 ± 2.41	0.0390*
CFS	4.73 ± 3.65	0.97 ± 1.44	0.0000*

*SSDE* Sjogren’s syndrome dry eye, *BCVA* best-corrected visual acuity, *TBUT* tear break-up time, *S1T* Schirmer test without anesthesia, *CFS* corneal fluorescein staining

* *p* < 0.05 statistically significant

### Comparison of VR-QoL scores for the SSDE and non-SSDE groups

NEI-VFQ subscales, including general health, general vision and long-distance vision activities, scored significantly lower (worse) in the SSDE group than in the non-SSDE group (all *p* values were <0.05). There were no statistically significant differences in subscales for ocular pain, short-distance vision activities, social function, mental health, role difficulties, dependency, driving, color vision, or peripheral vision (Table 2). The OSDI composite score for the SSDE group was higher (worse) than the non-SSDE group, although this was not significant (*p* = 0.1598). The SSDE group experienced significantly more severe ocular symptoms compared with the control group (*p* = 0.0256), whereas vision-related function and the effect of environmental triggers were not statistically significant (Table 2).

**Table 2:** NEI-VFQ and OSDI scores in the SSDE and non-SSDE groups

Scale	SSDE	Non-SSDE	Cohen’s d value	*P*-value
General health	34.2 ± 19.7	49 ± 21.7	0.7282	0.0075*
General vision	51 ± 20.8	63 ± 14.2	0.6862	0.0217*
Ocular pain	55.8 ± 25.4	57.9 ± 27.9	0.0894	0.7347
Short distance vision activities	73.67 ± 24.42	79.53 ± 24.05	0.2460	0.2293
Long distance vision activities	81.13 ± 20.42	92.19 ± 12.90	0.6591	0.0240*
Social function	90.69 ± 14.59	95.83 ± 8.96	0.4319	0.1317
Mental health	61.83 ± 29.02	63.17 ± 27.78	0.0477	0.9406
Role difficulties	66.67 ± 28.16	76.04 ± 24.57	0.3608	0.2518
Dependency	73.13 ± 26.57	77.71 ± 24.98	0.1808	0.4972
Driving	62.7 ± 30.37	65.4 ± 34.88	0.0840	0.5483
Color vision	91.67 ± 17.78	97.50 ± 10.06	0.4105	0.1447
Peripheral vision	90.83 ± 17.96	97.50 ± 7.63	0.4696	0.1411
Composite OSDI score	45.83 ± 23.62	36.32 ± 21.35	0.4296	0.1598
Ocular symptoms	49.83 ± 27.87	34.50 ± 23.79	0.6018	0.0256*
Vision-related function	29.58 ± 25.52	25.63 ± 26.58	0.1542	0.4613
Environmental Triggers	60.83 ± 34.40	53.61 ± 34.99	0.2116	0.3829

Effect size was determined by Cohen’s d value

*OSDI* ocular surface disease index, *NEI-VFQ* National Eye Institute Visual Function Questionnaire, *SSDE* Sjogren’s syndrome dry eye

* *p* < 0.05 statistically significant

### Comparison of psychological status in the SSDE and non-SSDE groups

Compared with the non-SSDE group, both the SAS and SDS scores of the SSDE group were higher (*p* = 0.0072 and 0.0162 respectively), as shown in Table 3. The prevalence of anxiety and depression symptoms in the SSDE group was significantly higher than in the control group (*p* = 0.0240 and 0.0200 respectively), as shown in Table 3.

**Table 3:** Comparison of psychological status in the SSDE and non-SSDE groups

	SSDE (*n* = 30)	Non-SSDE (*n* = 30)	T	Z	*P* value
SAS	50.1 ± 11.5	42.8 ± 7.4	2.7868	_	0.0072*
SDS	54.1 ± 12.7	46.4 ± 9.2	2.4760	_	0.0162*
Anxiety disorder, n (%)	13 (43.33%)	5 (16.67%)	_	5.0794	0.0240*
Depression disorder, n (%)	18 (60%)	9 (30%)	_	5.4545	0.0200*

*SSDE* Sjogren’s syndrome dry eye, *SAS* Zung Self-Rating Anxiety Scales, *SDS* Zung Self-Rating Depression Scales

* *p* < 0.05 statistically significant

### Correlations between VR-QOL scores, demographic characteristics, clinical parameters, and psychometric measures in the SSDE group

Spearman’s correlation analysis for the SSDE group revealed that the NEI-VFQ subscales for ocular pain and social function were both negatively correlated with CFS scores (both *p* values were <0.05), as shown in Table 4. Both the general vision and ocular pain NEI-VFQ subscales were positively correlated with TBUT values (both *p* values were <0.05), as shown in Tables 4. The 12 NEI-VFQ subscales were negatively correlated with the SAS/SDS scores (all *p* values were <0.05), as shown in Table 4, with the exception of social function, color vision, and peripheral vision. They were positively correlated with BCVA, with the exception of general health, general vision, and driving. The composite OSDI scores and its three subscale scores were positively correlated with the overall SAS/SDS scores (all *p* values were <0.05), as shown in Table 5. The composite OSDI score and its three subscale scores had a negative correlation with BCVA and TBUT, with the exception of the OSDI function subscale. They were positively correlated with CFS values (all *p* values were <0.05), as shown in Table 5. The overall SAS score had a correlation with the TBUT values (*p* = 0.0159) and CFS values (*p* = 0.0009). The overall SDS score was positively correlated with the CFS score (*p* = 0.0035). Neither the SAS nor the SDS scores were correlated with age, sex, household income levels, educational levels, duration of disease, or S1T (all *p* values were > 0.05), as seen in Table 6. In women with SSDE, regression analyses showed that the CFS score was a significant contributor for the NEI-VFQ visual pain subscale (standardized coefficient −3.973, 95% CI, −6.174 to −1.771, *p* = 0.001), the social functioning subscale (−1.707, 95% CI, −3.102 to −0.311; *p* = 0.018), and the OSDI ocular symptoms subscale (3.799, 95% CI, 1.242 to 6.357, *p* = 0.001). The TBUT value was found to be a significant contributor for total OSDI scores in SSDE women (standardized coefficient −5.159, 95% CI, −7.983 to −2.336, *p* = 0.001) and for the environmental trigger subscale (−7.848, 95% CI, −11.867 to −3.830, *p* = 0.000). The near vision (−0.226, 95% CI, −0.381 to −0.071, *p* = 0.006), mental health (−0.184, 95% CI, −0.310 to −0.059, *p* = 0.006), visual pain (0.264, 95% CI, 0.108 to 0.420, *p* = 0.002), and total OSDI scores (0.182, 95% CI, 0.029 to 0.335, *p* = 0.021) also made a significant contribution to the SAS score in women with SSDE. In addition, the near vision subscale (standardized coefficient −0.370, 95% CI, −0.531 to −0.210, *p* = 0.000) was a significant contributor to the SDS score in women with SSDE.

**Table 4:** Correlations of NEI-VFQ scores with Socio-demographic/clinical parameters/psychological status in the SSDE group

Scale	Age	Duration	BCVA	CFS	TBUT	S1T	SAS	SDS
General health	−0.4651*	0.0137	0.3535	−0.0768	0.0891	0.0382	−0.4996*	−0.4864*
General vision	−0.2912	−0.0291	0.2793	−0.2923	0.4171*	0.1817	−0.6748*	−0.5407*
Ocular pain	−0.1337	0.0986	0.4922*	−0.4928*	0.5070*	0.1748	−0.4432*	−0.4187*
Short distance vision activities	−0.5775*	0.0339	0.4599*	−0.2298	0.2098	0.1380	−0.7043*	−0.6781*
Long distance vision activities	−0.2929	0.0778	0.4148*	−0.2355	0.2463	0.2785	−0.6500*	−0.6662*
Social function	−0.1260	0.4209*	0.4050*	−0.3841*	0.2450	0.1289	−0.3310	−0.2582
Mental health	−0.3246	0.0825	0.5247*	−0.1288	0.1244	0.1605	−0.6396*	−0.5983*
Role difficulties	−0.1708	0.0990	0.6033*	−0.2371	0.1437	0.0726	−0.4334*	−0.4429*
Dependency	−0.3342	0.1843	0.5468*	−0.2385	0.1460	0.1308	−0.5478*	−0.6043*
Driving	−0.1863	0.1033	0.2311	0.1390	−0.0842	0.1193	−0.5601*	−0.5556*
Color vision	0.1276	0.1225	0.4007*	−0.2016	0.1991	0.0956	−0.2719	−0.2874
Peripheral vision	−0.0832	0.1144	0.5169*	−0.3457	0.3225	0.0846	−0.3591	−0.3545

*NEI-VFQ* National Eye Institute Visual Function Questionnaire, *SSDE* Sjogren’s syndrome dry eye, *SAS* Zung Self-Rating Anxiety Scales, *SDS* Zung Self-Rating Depression Scales, *TBUT* tear break-up time, *S1T* Schirmer test without anesthesia, *CFS* corneal fluorescein staining, *BCVA* best-corrected visual acuity

* *p* < 0.05 statistically significant

**Table 5:** Correlations of OSDI scores with demographic/clinical parameters/psychological status in the SSDE group

Scale	Age	Duration	BCVA	CFS	TBUT	S1T	SAS	SDS
Composite OSDI score	0.1471	0.0402	−0.4726*	0.4516*	−0.5513*	−0.2103	0.3296*	0.4083*
Ocular symptoms	0.1132	0.0468	−0.6078*	0.4477*	−0.4965*	−0.0814	0.4563*	0.5031*
Vision-related function	0.0372	0.0798	−0.199	0.3169	−0.3423	−0.0116	0.2467*	0.3107*
Environmental trigger	0.1006	0.1057	−0.3728*	0.4669*	−0.6217*	−0.254	0.5673*	0.6104*

*OSDI* Ocular Surface Disease Index, *SSDE* Sjogren’s syndrome dry eye, *SAS* Zung Self-Rating Anxiety Scales, *SDS* Zung Self-Rating Depression Scales, *BCVA* best-corrected visual acuity, *TBUT* tear break-up time, *S1T* Schirmer test without anesthesia, *CFS* corneal fluorescein staining

* *p* < 0.05 statistically significant

**Table 6:** Correlation of SAS/SDS scores with demographic and clinical parameters in SSDE patients

	SAS scores		SDS scores	
	*r*	*P* value	*r*	*P* value
Age	0.2187	0.0933	0.2223	0.0877
Educational level	−0.1597	0.2229	−0.1768	0.1756
Household income	−0.0787	0.7310	−0.0780	0.6429
Duration of disease	0.0325	0.8055	0.1107	0.3996
TBUT	−0.3102	0.0159*	−0.2192	0.0924
S1T	−0.0051	0.9692	−0.0281	0.8310
CFS	0.4639	0.0009*	0.3672	0.0035*
BCVA	−0.1296	0.3235	−0.2447	0.0595

*SSDE* Sjogren’s syndrome dry eye, *SAS* Zung Self-Rating Anxiety Scales, *SDS* Zung Self-Rating Depression Scales, *TBUT* tear break-up time, *S1T* Schirmer test without anesthesia, *CFS* corneal fluorescein staining, *BCVA* best-corrected visual acuity

* *p* < 0.05 statistically significant

## Discussion

The results of the NEI-VFQ and OSDI scores in this study showed a lower VR-QoL in women with SSDE compared with non-SSDE subjects. The NEI-VFQ subscales, including general health, general vision and long-distance vision activities, scored significantly lower (worse) in the SSDE group. Compared with peers, women with SS complained more of reduced wellbeing, fatigue, and arthralgia [24], which may have led to lower scores for general health. The SSDE patients showed more severe corneal epithelial damage than the non-SSDE patients in the current study, similar to the findings in a study by Liew et al. [25] The diminished function of the corneal epithelial barrier may be directly related to the effects of chronic inflammation in patients with SSDE [26, 27]. The integrity of the corneal epithelium is essential to the maintenance of tear film stability, whereas corneal epithelial defects may lead to irregularity in the refractive surface and tear film instability, and thus to optical interference and fluctuating functional visual acuity. Both of these conditions exert a negative impact on general and long-distance vision activities [28–30]. Unexpectedly, a strong correlation was found between NEI-VFQ/OSDI scores and BCVA in the SSDE group but not in the non-SSDE group, although BCVA in both groups did not show any significant differences. An explanation for this finding is that SSDE patients with normal BCVA (≥20/20) may have continued to complain of blurred vision because a decrease in contrast sensitivity at the peak frequency can have a significant effect on visual quality for SSDE patients [31].

In addition, the results of the SAS and SDS evaluation indicate significantly greater prevalence and severity of anxiety and depression in women with SSDE than in the non-SSDE group. In our previous work, we found that dry eye patients were more anxious and depressed than those without dry eye syndrome [32]. A previous study by Le et al. suggested that impairment of VR-QoL has a significant correlation with the severity of dry eye [17]. The SS patients in our study had more severe ocular symptoms and signs of dry eye than non-SSDE patients, which may lead to lower VR-QoL. SSDE patients tend to report more severe symptoms of anxiety and depression. This viewpoint is supported by our current results. First, psychological status (the SAS/SDS evaluation) was related to the perception of VR-QoL (NEI-VFQ/OSDI) in the SSDE group. This was also true for non-SSDE patients (data not shown). In addition, the overall SAS and SDS scores were positively correlated with CFS values in the SSDE group but not in the non-SSDE group. The regression analyses showed that the CFS score was a significant contributor for the NEI-VFQ visual pain and social functioning subscales, and the OSDI ocular symptoms subscale. TBUT values were found to be a significant contributor for the total OSDI score and the environmental trigger subscale in women with SSDE. The overall OSDI and NEI-VFQ subscales, including near vision, mental health and visual pain, were significant contributors for SAS scores. The near vision NEI-VFQ subscale makes a significant contribution to SDS scores in women with SSDE. The severity of CFS and TBUT showed a positive correlation with ocular pain and OSDI subscale scores in the SSDE group. Low vision quality and chronic ocular pain, which were closely related to corneal epithelial damage [28], may contribute to the occurrence of anxiety and depression symptoms. Many studies have reported that SS patients experience an increased rate of anxiety or depression [33–36]. A study by Valtysdottir et al. [35], using the hospital anxiety and depression scale (HADS), reported prevalence levels of anxiety and depression in 62 primary SS patients at 48 and 32%, respectively. In our study population of 30 SS patients, prevalence levels of anxiety and depression were 43.33 and 60%, respectively. These differences may have been the result of the different instruments used for measuring anxiety and depression and the small sample size of our study. As we know, depression and anxiety have adverse effects on a person’s work performance [37], interpersonal relationships [38], and general health [39]. In addition, they can also affect self-assessed disease severity and its treatment [40]. It is therefore essential to evaluate SSDE women who find no relief for ocular symptoms despite extensive use of palliative treatments using SAS/SDS questionnaires, and to refer them to a psychiatrist for in-depth consultation and broader care, including psychological intervention.

The current study found that the NEI-VFQ scores were negatively correlated with OSDI scores in SSDE participants. Previous studies have also demonstrated that NEI-VFQ scores have a moderate to strong correlation with OSDI scores among patients with SS and moderate-to-severe dry eye [41]. We therefore used the NEI-VFQ, a generic VR-QoL instrument, and the OSDI, a dry eye–specific instrument, to gain the full scope of information on patient status. The combination of generic and disease-specific questionnaires gave a more comprehensive picture of VR-QoL. In terms of limitations, the sample size of the two groups was small. A larger sample size from a multi-center approach is therefore required to confirm these findings. As contrast sensitivity is the single best marker of functional visual acuity, further studies should be conducted to apply contrast sensitivity to investigate the association between vision quality and the VR-QoL of SSDE patients. The questionnaire survey was conducted after the ocular examinations, which may have had an impact on the feelings of the patients. Although the patients had sufficient time (about 40 min) to recover from the effects of the contact inspections, more reliable results may be obtained if questionnaires are administrated before ocular examinations. We nevertheless consider our findings to be of great significance because they provide information on the differences in quality of life between SSDE and non-SSDE patients. Information on the major determinants of VR-QoL is necessary to select appropriate management strategies for women with SSDE. The present study found that corneal epithelial damage and tear film instability in women with SSDE made a significant contribution to the impairment of VR-QoL, indicating that targeted treatment to improve TBUT and CFS in SSDE subjects could help improve their VR-QoL. We believe the relationship between psychological status and VR-QoL to be bidirectional. Patients who remained in a better psychological state had a better VR-QoL, and patients who had a better VR-QoL were less likely to experience symptoms of anxiety or depression. Therefore, when treating women with SSDE, an evaluation for symptoms of anxiety and depression is essential for the provision of optimal care.

## Conclusions

In conclusion, we believe that SSDE is associated with a more adverse impact on VR-QoL than the case with non-SSDE patients. The impairment of VR-QoL in SSDE patients has a significant impact on the psychological disturbances they experience.

## Abbreviations

BCVA: Best-corrected visual acuity
CFS: Corneal fluorescein staining
NEI-VFQ: National Eye Institute Visual Function Questionnaire
OSDI: Ocular Surface Disease Index
S1T: Schirmer1 test without anesthesia
SAS: The Zung Self-Rating Anxiety Scales
SDS: The Zung Self-Rating Depression Scales
SS: Sjogren’s syndrome
SSDE: Sjogren syndrome dry eye
TBUT: Tear break-up time
VR-QoL: Vision-related quality of life
